# Sexual Dimorphism of Gut Microbiota Dictates Therapeutics Efficacy of Radiation Injuries

**DOI:** 10.1002/advs.201901048

**Published:** 2019-08-29

**Authors:** Ming Cui, Huiwen Xiao, Yuan Li, Shuqin Zhang, Jiali Dong, Bin Wang, Changchun Zhu, Mian Jiang, Tong Zhu, Junbo He, Haichao Wang, Saijun Fan

**Affiliations:** ^1^ Tianjin Key Laboratory of Radiation Medicine and Molecular Nuclear Medicine Institute of Radiation Medicine Chinese Academy of Medical Sciences and Peking Union Medical College 238 Baidi Road Tianjin 300192 China; ^2^ Laboratory of Emergency Medicine Feinstein Institute for Medical Research Manhasset NY 11030 USA

**Keywords:** adverse side effects, gut microbiota, radiotherapy, sexual dimorphism

## Abstract

Accidental or iatrogenic ionizing radiation exposure precipitates acute and chronic radiation injuries. The traditional paradigm of mitigating radiotherapy‐associated adverse side effects has ignored the gender‐specific dimorphism of patients' divergent responses. Here, the effects of sexual dimorphism on curative efficiencies of therapeutic agents is examined in murine models of irradiation injury. Oral gavage of simvastatin ameliorates radiation‐induced hematopoietic injury and gastrointestinal tract dysfunction in male mice, but adversely deteriorates these radiation syndromes in female animals. In a sharp contrast, feeding animals with high‐fat diet (HFD) elicites explicitly contrary results. High‐throughput sequencing of microbial 16S rRNA, host miRNA, and mRNA shows that simvastatin or HFD administration preventes radiation‐altered enteric bacterial taxonomic structure, preserves miRNA expression profile, and reprogrammes the spectrum of mRNA expression in small intestines of male or female mice, respectively. Notably, faecal microbiota transplantation of gut microbes from opposite sexual donors abrogates the curative effects of simvastatin or HFD in respective genders of animals. Together, these findings demonstrate that curative efficiencies of therapeutic strategies mitigating radiation toxicity might be dependent on the gender of patients, thus simvastatin or HFD might be specifically useful for fighting against radiation toxicity in a sex‐dependent fashion partly based on sex‐distinct gut microbiota composition in preclinical settings.

## Graphical abstract

**Traditional remedies for radiotherapy‐intertwined adverse side effects** ignore the sex‐specific dimorphism of patients' divergent responses. Individuals of different sexes represent variant performances following a same therapeutic regimen. In the present study, simvastatin or high fat diet is developed to specifically protect against radiation toxicity of males or females partly based on sex‐distinct gut microbiota structure.

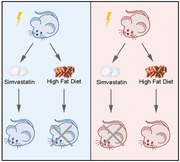

## 1 Introduction

Cancer has gradually become a leading cause of death throughout the world. Approximately half of all cancer diagnoses have an indication for radiotherapy, with substantially greater use in the metastatic settings.^1^, ^2^ After radiation exposure, a complex spectrum of clinical complications are intertwined, including bone marrow toxicity (hematopoietic syndrome) and gastrointestinal toxicity (GI syndrome), which are collectively known as acute radiation syndrome (ARS).^3^, ^4^ Owing to the sensitivity of hematopoietic system and GI tract toward ionizing radiation, ARS facilitates intractable pathologic processes and may even cause death.^5^, ^6^ To date, with the on‐going elevation in the number of cancer survivors, prevention of radiotherapy‐associated adverse side effects has become an urgent priority.^7^, ^8^ Even for the healthy population, unwanted radiation exposure, such as terrorist events, industrial accidents, and natural disasters, in a mass casualty setting is also a serious public health concern.^9^ Heretofore, curative therapeutic approaches to mitigate radiation toxicity remain unmet and urgent medical needs.

Mammalian GI tract harbors trillions of symbiotic microbes within the lumen collectively named as gut microbiota which shaped by common factors including diet, lifestyle, medication, early‐life determinants, environment, and genetics.^10^, ^11^ Recently, investigations focusing on gut microbiota have experienced a renaissance, and mounting evidence underpins essential roles of enteric microbes as key regulatory elements in physiologic and pathologic status of hosts.^12^, ^13^, ^14^ For instance, gut microbiota governs metabolic process and energy balance, and educates the innate and adaptive immune responses of hosts.^15^, ^16^ Our group has proved that radiation exposure shapes the abundance and composition of gut microbiota,^6^, ^17^ and fecal microbiota transplantation (FMT) of gut microbes from healthy donor may provide a therapeutic strategy for radiation‐caused toxicity.^18^ However, interindividual variations in the gut microbiome might be potential pitfalls during FMT performance enmeshed multiple pathologies.^19^ Importantly in the previous study, we uncovered that the gut microbiota structure between male and female mice exhibited a clear dimorphism in both microbial abundance and composition.^18^

Sexual dimorphism widely exists throughout all animal kingdom.^20^ Sex differences in behavior arise from sexual dimorphism in the underlying neural circuits.^21^, ^22^ Sexual dimorphism in immune function is common in vertebrates and even in a number of invertebrates,^23^ manifested commonly as females being more “immunocompetent” than males.^24^ Traditional pharmacological and medical studies have considered male and female organisms as equivalent, and most preclinical and clinical research was carried out in one sex (mainly males) and the results extrapolated to the other which dramatically limits the impact of research findings. For instance, although FMT mitigates radiation‐induced toxicity, the sex difference between donors and recipients indeed influences the curative effects. Moreover, even if a phenotype does not exhibit an overall sex difference, underlying mechanisms may still differ in the two sexes.^25^ Thus, whether therapeutic approaches for radiation‐caused toxicity representing sexual dimorphism remains poorly understood, which possesses pivotal clinical value. As a HMG‐CoA reductase inhibitor and an anticholesterol drug, simvastatin has also been reported to mitigate radiation toxicity.^26^ However, the evolution of males and females facilitates comprehension of sex‐specific physiology and, consequently, differential susceptibility to diseases with particular reference to those involving energy metabolism.^27^ Especially in lipid metabolism, high fat diet (HFD) induces adipogenesis in visceral white adipose tissue in male mice, while in females HFD induces adipogenesis in both visceral white adipose tissue and subcutaneous adipose tissue.^28^ Accordingly, whether simvastatin and HFD representing sexual dimorphism in radioprotection remain enigmas.

In the present study, we report that gender‐specific differences impact the treatment efficacies toward radiation‐induced toxicity and further evaluate the underlying molecular mechanisms in mouse models. To mimic accidental irradiation and radiation therapy to pelvic and abdominal cancers, the male and female mice were exposed to 7 Gy total body irradiation to assess survival rate, 4 Gy total body irradiation to assess hematopoietic toxicity, and 12 Gy total abdominal irradiation to assess GI toxicity. Overall, administration of simvastatin by oral route is medicative for irradiated male mice, while HFD is specifically protective only for females. Simvastatin manipulation and HFD consumption restored the radiation‐induced derangement of intestinal bacterial taxonomic pattern, reprogramed the gene expression profile of small intestine tissues in respective male and female mice. Thus, our findings provide new insights into the therapeutic strategies for radiotherapy‐associated adverse effects based on gender distinction and dissect the underlying protective mechanism. Our observations support that treatments for acute radiation syndrome should consider sexual dimorphism in both experimental and clinical settings.

## 2 Results

### 2.1 Therapeutic Strategy toward Radiation‐Caused Toxicity Relates to Sexual Distinction

**Figure 1:**
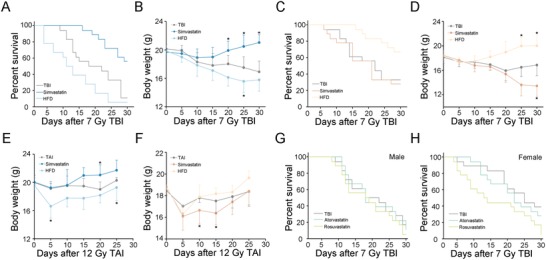
Sexual distinction impacts therapeutic effects toward radiation‐caused toxicity. A,B) Kaplan–Meier analysis A) and body weight measurement B) of male mice in the three groups after 7 Gy TBI, *n* = 30 per group; *P* < 0.05 by log‐rank test between simvastatin‐treated group and control (A); *P < 0.05 by Student's *t*‐test between simvastatin‐treated/HFD group and control (B). C,D) Kaplan–Meier analysis C) and body weight measurement D) of female mice in the three groups after 7 Gy TBI, *n* = 30 per group; *P* < 0.05 by log‐rank test between HFD group and control (C); *P < 0.05 by Student's *t*‐test between simvastatin‐treated/HFD group and control (D). E,F) Body weight was compared among three group mice of male E) and female F) after 12 Gy TAI, *n* = 24 per group; *P < 0.05 by Student's *t*‐test between simvastatin‐treated/HFD group and control. G,H) Kaplan‐Meier analysis of male G) and female H) mice treated with atorvastatin and rosuvastatin after 7 Gy TBI, *n* = 24 per group.

Male and female mice were orally given simvastatin or fed with high‐fat diet (HFD) following 7 Gy total body irradiation (TBI). The simvastatin group exhibited a higher survival rate and body weight in male mice, but HFD led to a poorer state in these male animals (**Figure** **1**A,B). On the contrary, HFD treatment, but not simvastatin, elevated survival rate and reduced the weight loss of female mice (Figure 1C,D). In 12 Gy total abdominal irradiation (TAI) models, HFD similarly decreased the body weight of male mice but increased that of female mice, which was in a sharp contrast with the efficacy of simvastatin (Figure 1E,F), indicating a gender‐specific and distinct response to two radiation therapeutic agents in two animal models of radiation injury. Atorvastatin and rosuvastatin failed to impact the survival rate and body weight of male or female mice after radiation exposure (Figure 1G,H; Figure S1A,B, Supporting Information), suggesting simvastatin as a specific and therapeutic statin for radiation injury in male animals.

### 2.2 Effects of Simvastatin and HFD on Hematopoietic System Injury of Irradiated Male and Female Mice

**Figure 2:**
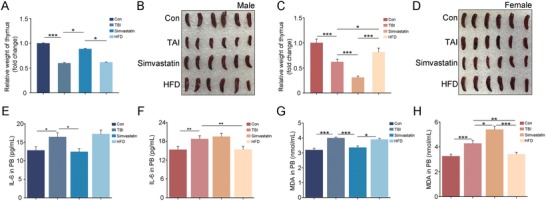
Simvastatin or HFD ameliorates radiation‐associated hematopoietic syndrome of male or female mice. A,B) Volume of dissected thymuses A) and Photographs of spleens B) from male mice in the four groups, the thymuses and spleens were obtained at day 21 after 4 Gy TBI. Mean ± SD. Significant differences are indicated: *P < 0.05, ***P < 0.005 by Student's *t*‐test between each two cohort, *n* = 18 per group. C,D) Volume of dissected thymuses C) and photographs of spleens D) from female mice in the four groups, the thymuses and spleens were obtained at day 21 after 4 Gy TBI. Mean ± SD. Significant differences are indicated: *P < 0.05, ***P < 0.005 by Student's *t*‐test between each two cohort, *n* = 18 per group. E,F) The levels of IL‐6 in PB of male E) and female F) were examined. Significant differences are indicated: *P < 0.05, **P < 0.01 by Student's *t*‐test between each two cohort, *n* = 18 per group. G,H) The levels of MDA in PB of male G) and female H) were examined. Significant differences are indicated: *P < 0.05, **P < 0.01, ***P < 0.005 by Student's *t*‐test between each two cohort, *n* = 18 per group.

Following total body irradiation, both male and female mice carried atrophied thymuses and spleens, indicating irradiation‐induced hematopoietic system toxicity (**Figure** **2**A–D; Figure S2A,B, Supporting Information). Oral gavage of simvastatin restored thymus and spleen tissues in male mice; whereas HFD conferred a similar protection only in female animals (Figure 2A–D; Figure S2A,B, Supporting Information). In a sharp contrast, simvastatin administration reduced the weight of thymuses and spleens in irradiated female mice; whereas HFD did not alter the weight of these tissues in male animals, implying that simvastatin might be a detrimental agent for female patients receiving radiotherapy (Figure 2A–D; Figure S2A,B, Supporting Information). Simvastatin erased the radiation‐heightened IL‐6 and TNFɑ in peripheral blood (PB) of irradiated male mice (Figure 2E; Figure S3A, Supporting Information); whereas HFD abrogated those changes in irradiated female animals (Figure 2F; Figure S3B, Supporting Information). The alterations of malonaldehyde (MDA) in PB were coincident with those of inflammation factors (Figure 2G, H). Together, our observations demonstrate that simvastatin might be a therapeutic option toward radiation‐associated hematopoietic system toxicity for male animals. For female, HFD might be employed to protect against hematopoietic system injury induced by irradiation.

### 2.3 Sexual Distinction Governs Rehabilitation Efficacy of Simvastatin and HFD toward Radiation‐Induced Gastrointestinal Toxicity

**Figure 3:**
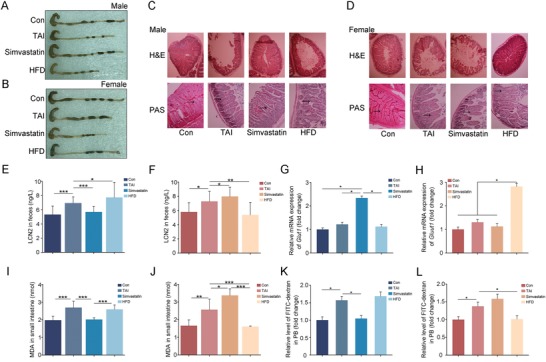
Rehabilitation efficacy of simvastatin and HFD toward radiation‐induced gastrointestinal toxicity relates to sexual dimorphism. Male and Female mice were exposed to 12 Gy total abdominal irradiation, the colon and small intestine tissues were obtained at day 21, *n* = 24 per group. A,B) Photographs of dissected colon from male A) and female B) mice in the four groups. C,D) The morphology of the small intestine form male C) and female D) mice was shown by H&E and PAS staining. The black arrows point to the goblet cells. E,F) The levels of LCN2 in fecal pellets from male E) and female F) mice were examined by ELISA. Significant differences are indicated: *P < 0.05, **P < 0.01, ***P < 0.005 by Student's *t*‐test between each two cohort. G,H) The expression levels of *Glut1* are examined in small intestine tissues from male G) and female H) mice by quantitative PCR. Significant differences are indicated: *P < 0.05 by Student's *t*‐test between each two cohort. I,J) The levels of MDA were assessed in small intestine tissue from male I) and female J) mice. Significant differences are indicated: *P < 0.05, **P < 0.01, ***P < 0.005 by Student's *t*‐test between each two cohort. K,L) The levels of FITC‐dextran in PB from male K) and female L) mice were assessed. Significant differences are indicated: *P < 0.05 by Student's *t*‐test between each two cohort.

Next, we performed TAI to mimic radiotherapy toward abdominal and pelvic malignancies. Both male and female mice carried shorter colons implying possible intestinal inflammation, fewer intact intestinal villi and goblet cells representing disruption of small intestinal epithelial integrity after 12 Gy TAI (**Figure** **3**A–D; Figure S4A,B, Supporting Information). As expected, simvastatin or HFD mitigated the irradiated impairment of male or female mice respectively; however, male mice received HFD or female mice gained simvastatin represented serious GI toxicity (Figure 3A–D; Figure S4A,B, Supporting Information). At the molecular level, simvastatin or HFD treatment erased the elevation IL‐6 and TNFɑ in small intestine tissues (Figure S5A–D, Supporting Information), and LCN2 in fecal pellets from abdominal irradiated male or female mice (Figure 3E,F). But the levels of LCN2 further increased when male mice treated with HFD or female mice treated with simvastatin (Figure 3E,F), suggesting that simvastatin and HFD alleviate radiation‐caused intestinal inflammation in a sexual‐dependent fashion. Simvastatin or HFD treatment up‐regulated the expression of *Glut1* (*Slc2a1*), multidrug resistance protein 1 (*MDR1*), and *Pgk1*, which participate in epithelial integrity maintaining after toxic stimuli, in the small intestine tissues from abdominal irradiated male or female mice (Figure 3G,H; Figure S6A–D, Supporting Information). Simvastatin or HFD administration also reduced the radiation‐heightened MDA and expression of *Nrf2* in irradiated male or female mice (Figure 3I,J; Figure S7A,B, Supporting Information), indicating that simvastatin and HFD might be employed to fight against radiation‐induced production of cytotoxic reactive oxygen species in a sex‐dependent manner. To further validate simvastatin or HFD treatment improves GI tract function and epithelial integrity in abdominal irradiated male or female animals, we confirmed that simvastatin and HFD administration eliminated radiation‐elevated FITC‐dextran levels in PB (Figure 3K,L).

### 2.4 Gut Microbiota Structure Emerges a Sex‐Biased Response to Distinct Therapeutic Approaches

**Figure 4:**
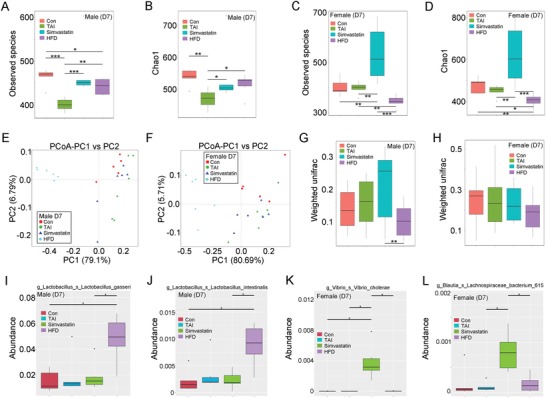
Oral gavage of simvastatin and HFD educate radiation‐shifted intestinal bacterial structure at day 7 after TAI based on the gender of animal. For box plot, the top and bottom boundaries of each box indicate the 75th and 25th quartile values, respectively, and lines within each box represent the 50th quartile (median) values. Ends of whiskers mark the lowest and highest diversity values in each instance. A,B) The observed species number A) and Chao1 diversity index B) of intestinal bacteria in male mice was examined by 16S rRNA high‐throughput sequencing after 7 d of TAI exposure. Significant differences are indicated: Wilcoxon rank sum test. *n* = 5 (control group) or 6 (irradiated groups). C,D) The observed species number C) and Chao1 diversity index D) of intestinal bacteria in female mice was examined by 16S rRNA high‐throughput sequencing after 7 d of TAI exposure. Significant differences are indicated: Wilcoxon rank sum test. *n* = 5 (control group) or 6 (irradiated groups). E,F) Principle coordinate analysis (PCoA) were performed to assess the shift in intestinal bacterial composition profile from male E) and female F) mice after irradiation at day 7. *n* = 5 (control group) or 6 (irradiated groups). G,H) The β diversity of intestinal bacteria was compared by weighted unifrac analysis. Significant differences are indicated: Wilcoxon rank sum test. *n* = 5 (control group) or 6 (irradiated groups). I–L) The abundances of most varied strain bacteria in male I, J) and female K,L) mice was assessed using 16S high‐throughput sequencing after irradiation at day 7. Significant differences are indicated: Wilcoxon rank sum test. *n* = 5 (control group) or 6 (irradiated groups).

To further validate simvastatin and HFD can be employed in clinical tracts to fight against side effects of male and female patients with pelvic and abdominal cancers after radiotherapy, we focused on the underlying mechanism by which simvastatin and HFD ameliorate GI injury. Given gut microbiota related to radiation injury of hosts,^29^ we assessed the enteric bacteria taxonomic proportions of male and female mice following radiation exposure. At day 7 after TAI, male mice harbored fewer observed species number of gut bacteria, simvastatin manipulation and HFD consumption erased the alterations (**Figure** **4**A,B; Figure S8A, Supporting Information). For female, in overt opposition to simvastatin administration, HFD decreased the observed species number of enteric bacteria after TAI (Figure 4C,D; Figure S9A, Supporting Information). Weighted principle coordinate analysis (PCoA) and principal component analysis (PCA) based on significant difference at the genus level revealed that HFD consumption promoted different changes in bacterial taxonomic composition structure in male versus female mice (Figure 4E,F; Figures S8B,9B, Supporting Information). Statistically however, although weighted unifrac analysis showed no difference among the four cohorts of female, HFD drove a significant shift in gut flora composition of male mice (Figure 4G,H). At day 7 after TAI, simvastatin and HFD screened out diverse dominant bacteria in male and female mice (Figures S8C,9C, Supporting Information). In detail, irradiated male mice housing with HFD harbored higher abundance of *Lactobacillus gasseri* and *Lactobacillus intestinalis* at the species level (Figure 4I,J), whereas irradiated female mice with oral gavage of simvastatin carried higher abundance of *Vibrio cholerae* and *Lachnospiraceae bacterium* at the species level (Figure 4K,L).

**Figure 5:**
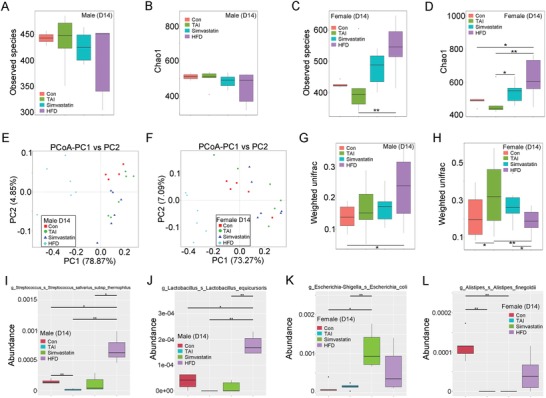
Simvastatin and HFD administration shape intestinal bacterial structure at day 14 after TAI in a sexual‐dependent fashion. A,B) The observed species number A) and Chao1 diversity index B) of enteric bacteria in male mice was measured by 16S rRNA high‐throughput sequencing at 14 d after TAI exposure. Significant differences are indicated: Wilcoxon rank sum test. *n* = 5 (control group) or 6 (irradiated groups). C,D) The observed species number C) and Chao1 diversity index D) of enteric bacteria in female mice was measured by 16S rRNA high‐throughput sequencing at 14 d after TAI exposure. Significant differences are indicated: Wilcoxon rank sum test. *n* = 5 (control group) or 6 (irradiated groups). E,F) PCoA were performed to assess the alteration of gut bacteria taxonomic profile from male E) and female F) mice after TAI at day 14. *n* = 5 (control group) or 6 (irradiated groups). G,H) The β diversity of intestinal bacteria was compared by weighted unifrac analysis. Significant differences are indicated: Wilcoxon rank sum test. *n* = 5 (control group) or 6 (irradiated groups). I–L) The abundances of most varied strain bacteria in male I,J) and female K,L) mice was examined using 16S high‐throughput sequencing after TAI at day 14. Significant differences are indicated: Wilcoxon rank sum test. *n* = 5 (control group) or 6 (irradiated groups).

At day 14 after TAI, no significant change in observed species number of enteric bacteria was observed among male mice from the four groups (**Figure** **5**A,B; Figure S10A, Supporting Information). In female mice however, HFD and simvastatin elevated the observed species number of intestinal bacteria (Figure 5C,D; Figure S11A, Supporting Information). PCoA and PCA plot based on significant difference at the genus level revealed an overt separation representing the shifted bacterial taxonomic composition of male and female mice in this investigation (Figure 5E,F; Figures S10B, 11B, Supporting Information). Statistics analysis further indicated that HFD educated enteric microbiota pattern of irradiated male mice (Figure 5G), but in female mice, HFD abrogated the alterations of intestinal microbiota composition drove by TAI (Figure 5H). Dominant bacteria in male and female mice under different condition for 14 d were distinct (Figures S10C, 11C, Supporting Information). Specifically, simvastatin administration erased the altered abundance of *Lactobacillus equicursoris* and *Streptococcus salivarus subsp thermophilus* in irradiated male mice at the species level (Figure 5I,J). HFD altered that of *Escherichia coli* (or *Alistipes finegoldii*) in irradiated female mice at the species level (Figure 5K,L).

### 2.5 The Optimal Therapeutic Options Reprogram Gene Expression Profile of Irradiated Mice Small Intestine

**Figure 6:**
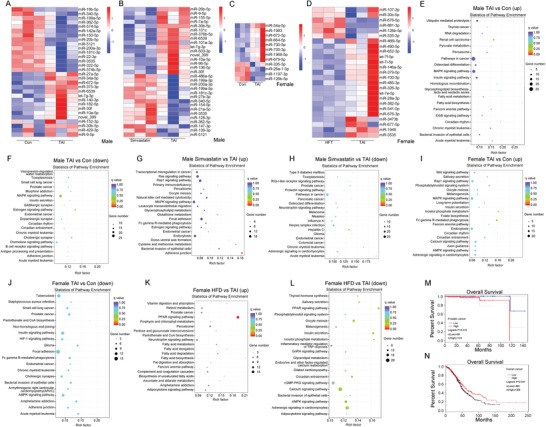
The optimal therapeutic options reprogram radiation‐shaped gene expression profile of mice small intestines. In this experiment, male mice were divided into three groups. One group was exposed to 12 Gy TAI, one group was treated with simvastatin following 12 Gy TAI exposure, another group was control. Female mice were also divided into three groups. One group was exposed to 12 Gy TAI, one group was fed with HFD following 12 Gy TAI exposure, another group was control. Small intestine tissues were obtained at day 21 after irradiation. A–D) Cluster analysis of differentially expressed miRNA of small intestine tissues from male A,B) and female C,D) mice. A: control group versus TAI group, B: simvastatin group versus TAI group, C: control group versus TAI group, D: HFD group versus TAI group. E–L) Bioinformatics analysis of different proteins clustered into pathways of small intestine tissues from male and female mice through KEGG. M,N) Kaplan–Meier analysis of overall survival rate of prostatic cancer and ovarian cancer patients. *P* < 0.05 by log‐rank test between the patients with high and low expression of HMGCR.

To address the molecular mechanism by which optimal therapeutic strategies ameliorating radiation‐induced toxicity in a sex‐dependent manner, we assessed the miRNA expression profile of TAI‐exposed mice with or without optimal treatments using sequencing technique. The miRNA expression profiles of small intestine tissues from male and female mice were changed following 12 Gy TAI (Figure S12A–D, Supporting Information). However, simvastatin manipulation via oral route or HFD reprogramed the miRNA expression profiles of irradiated male or female mice individually. Notably, TAI exposure up‐regulated the expression of let‐7g‐3p, miR‐30f, miR‐10a‐5p, miR‐9‐5p, miR‐6539, and novel_399, down‐regulated that of miR‐340‐5p, miR‐199a‐5p, miR‐362‐5p, miR‐5121, miR‐200a‐3p, miR‐181c‐3p, and miR‐3535 in small intestine tissues from irradiated male mice, which were reversed by oral gavage of simvastatin (**Figure** **6**A,B). In TAI‐exposed female mice, small intestine tissues carried higher level of miR‐700‐3p, lower level of miR‐126b‐5p and miR‐335‐3p, which were erased by feeding with HFD (Figure 6C,D), indicating that optimal therapeutic strategies retain the miRNA expression profile shifted by TAI.

We also examined the mRNA expression profile in this study. Volcano plot validated the alterations of mRNA expression profile mediated by TAI exposure with or without simvastatin administration or HFD in small intestine tissues from male or female victims (Figure S13A–D, Supporting Information). Comparing with the irradiated male mice treated with simvastatin, the small intestinal mRNA expression pattern of irradiated female mice feeding with HFD was more similar to that of control (Figure S13E,F, Supporting Information), implying that both simvastatin and HFD indeed educated the enteric mRNA expression profile in irradiated male and female mice. Finally, kyoto encyclopedia of genes and genomes (KEGG) analyses were performed to cluster the drifted genes drove by TAI with or without optimal therapy. In male mice, TAI activated the pathways involved in metabolism, such as insulin signaling and fatty acid biosynthesis, inhibited the pathways covering adherens junction and estrogen signaling. However, simvastatin treatment reactivated the pathway about adherens junction and estrogen signaling, and blocked the pathways implicated in carcinogenesis (Figure 6E–H). In female mice, TAI also restrained the pathways about adherens junction, importantly, HFD restructured the metabolic pattern of irradiated mice, such as activating fatty acid metabolism and dampening insulin secretion (Figure 6I–L), which were in the line with our previous study.^18^ These findings provided substantial evidence underpinning that although the symptoms of male and female mice after irradiation exposure were dissimilar, alleviating radiation‐induced toxicity should base on sexual dimorphism. In analogy to male mice, TAI disturbed the pathways involved in circadian rhythm of female mice (Figure 6E,I). Given statins are inhibitors of HMG‐CoA reductase (HMGCR),^30^ we analyze the overall survival rate of prostatic cancer and ovarian cancer patients based on the expression of HMGCR. Intriguingly, prostatic cancer patients with low level of HMGCR (http://gepia.cancer-pku.cn/) and ovarian cancer patients with high level of HMGCR (http://kmplot.com/analysis/) represented higher overall survival rate (Figure 6M,N).

### 2.6 Gut Microbiota Governs the Curative Effects of Simvastatin and HFD

**Figure 7:**
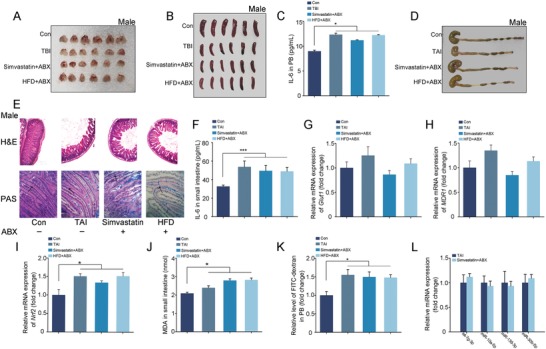
Gut microbiota contributes to the radioprotective effects of simvastatin and HFD in male mice. The mice were housed with antibiotic mixture (ABX) in drinking water. A,B) Photographs of thymuses A) and spleens B) from male mice in the four groups, the thymuses and spleens were obtained at day 21 after 4 Gy TBI. *n* = 18 per group. C) The level of IL‐6 in PB was examined. Significant differences are indicated: *P < 0.05 by Student's *t*‐test between each two cohort, *n* = 18 per group. D) Photograph of dissected colon from male mice in the four groups. E) The morphology of the small intestine from male mice was shown by H&E and PAS staining. The black arrows point to the goblet cells. F) The level of IL‐6 in small intestine tissues was examined by ELISA. Significant differences are indicated: *P < 0.05 by Student's *t*‐test between each two cohort, *n* = 18 per group. G–I) The expression levels of *Glut1* G), *MDR1* H), and *Nrf2* I) were examined in small intestine tissues by quantitative PCR. Significant differences are indicated: *P < 0.05 by Student's *t*‐test between each two cohort, *n* = 18 per group. J) The level of MDA was assessed in small intestine tissue. Significant differences are indicated: *P < 0.05 by Student's *t*‐test between each two cohort, *n* = 18 per group. K) The level of FITC‐dextran in PB was assessed. Significant differences are indicated: *P < 0.05 by Student's *t*‐test between each two cohort, *n* = 18 per group. L) The expression levels of miRNAs were examined in small intestine tissues by quantitative PCR. Significant differences are indicated by Student's *t*‐test between each two cohort, *n* = 18 per group.

**Figure 8:**
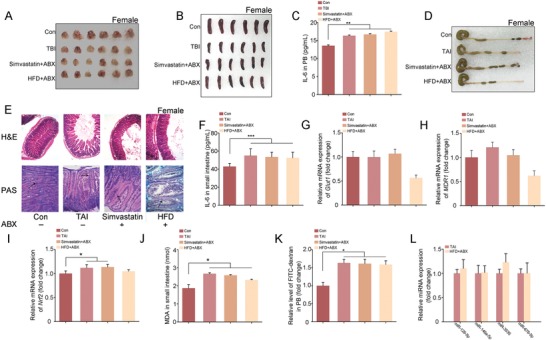
Gut microbiota contributes to the radioprotective effects of simvastatin and HFD in female mice. The mice were housed with antibiotic mixture (ABX) in drinking water. A,B) Photographs of thymuses A) and spleens B) from female mice in the four groups, the thymuses and spleens were obtained at day 21 after 4 Gy TBI. *n* = 18 per group. C) The level of IL‐6 in PB was examined. Significant differences are indicated: *P < 0.05 by Student's *t*‐test between each two cohort, *n* = 18 per group. D) Photograph of dissected colon from female mice in the four groups. E) The morphology of the small intestine from female mice was shown by H&E and PAS staining. The black arrows point to the goblet cells. F) The level of IL‐6 in small intestine tissues was examined by ELISA. Significant differences are indicated: *P < 0.05 by Student's *t*‐test between each two cohort, *n* = 18 per group. G–I) The expression levels of *Glut1* G), *MDR1* H), and *Nrf2* I) were examined in small intestine tissues by quantitative PCR. Significant differences are indicated: *P < 0.05 by Student's *t*‐test between each two cohort, *n* = 18 per group. J) The level of MDA was assessed in small intestine tissue. Significant differences are indicated: *P < 0.05 by Student's *t*‐test between each two cohort, *n* = 18 per group. K) The level of FITC‐dextran in PB was assessed. Significant differences are indicated: *P < 0.05 by Student's *t*‐test between each two cohort, *n* = 18 per group. L) The expression levels of miRNAs were examined in small intestine tissues by quantitative PCR. Significant differences are indicated by Student's *t*‐test between each two cohort, *n* = 18 per group.

Given gut microbiome interactions with drug metabolism, efficacy, and toxicity, the mice were housed with antibiotic mixture (ABX) in drinking water to eliminate enteric microorganisms. After simvastatin or HFD administration, ABX‐challenged male and female mice carried atrophic thymuses and spleens (**Figures** **7**A,B and **8**A,B; Figures S14A,B and S15A,B, Supporting Information), elevatory hematic IL‐6, and TNFɑ (Figures 7C, 8C; Figures S14C, S15C, Supporting Information) following TBI exposure, indicating that the radioprotection of simvastatin and HFD to hematopoietic system might be partly dependent on gut microbes. In addition, simvastatin and HFD also failed to mitigate radiation‐induced GI tract toxicity in ABX‐challenged male and female mice, representing as shorter colons (Figures 7D, 8D; Figures S14D, S15D, Supporting Information), fewer intact intestinal villi and goblet cells (Figures 7E, 8E), higher inflammatory factor levels in small intestine (Figures 7F, 8F; Figures S14E, S15E, Supporting Information), worse epithelial integrity (Figures 7G,H and 8G,H; Figures S14F, S15F, Supporting Information), and serious cytotoxic reactive oxygen species as well as cacoethic GI tract function (Figures 7I–K, 8I–K) compared with the mice without irradiation. Given gut microbes hijack hosts' miRNA profile,^31^, ^32^ we assessed the expression of several simvastatin‐modulated (or HFD‐modulated) miRNAs in small intestine tissues from ABX‐treated male (or female) mice following TAI exposure. Of note, simvastatin and HFD administration unchanged the expression of the miRNAs in small intestine without intestinal microflora (Figures 7L, 8L), indicating that gut microbiome might be the key elements governing the responses of hosts' intestinal miRNA profile to external stimulus, such as simvastatin and HFD.

**Figure 9:**
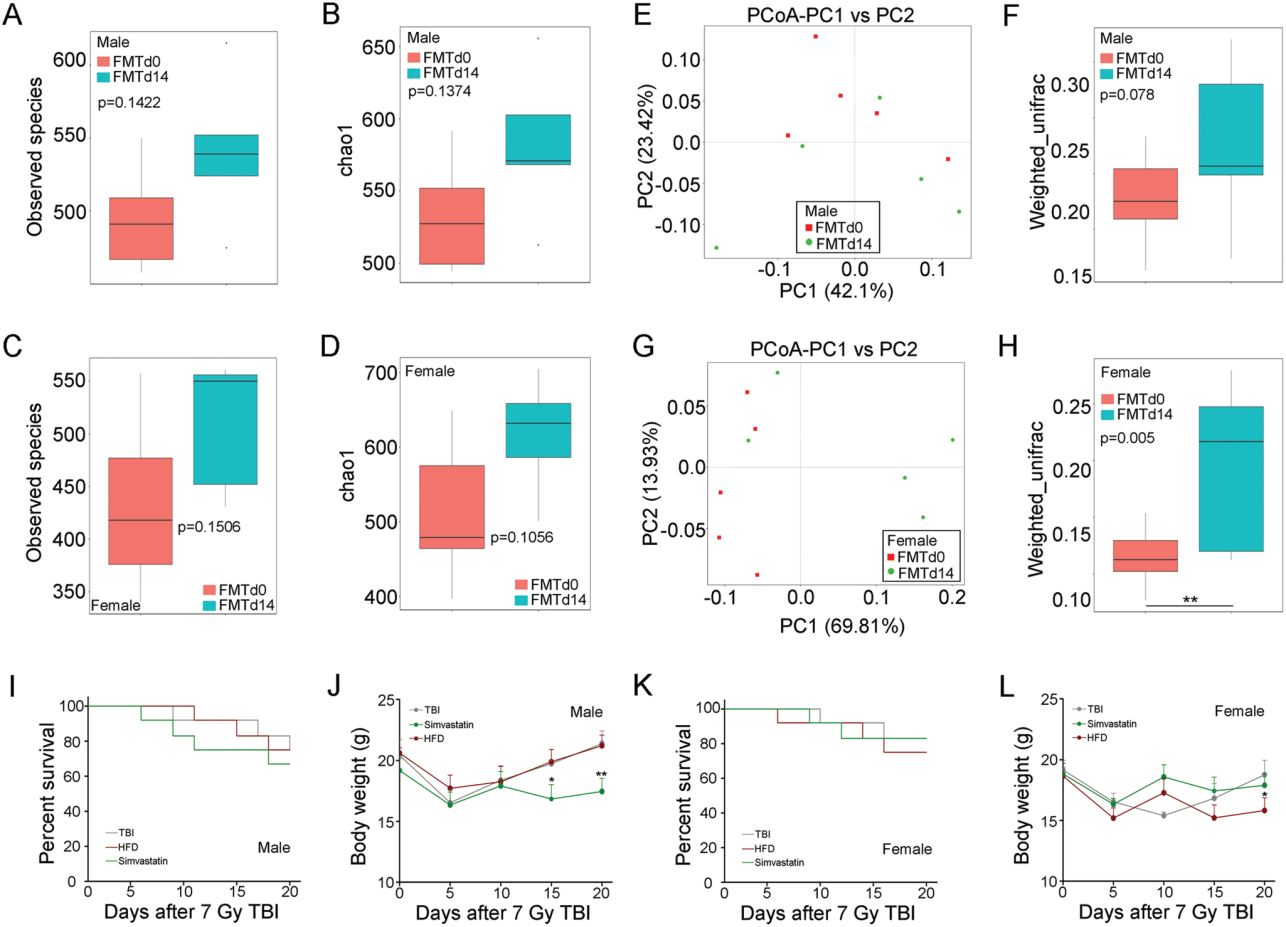
The curative effects of simvastatin and HFD mitigating radiation toxicity base on gut microbiota composition pattern. FMT was performed to male (or female) mice using fecal pellets from female (or male) mice for 14 d. Before 7 Gy TBI exposure, the fecal pellets of recipients were collected and assessed measured by 16S rRNA high‐throughput sequencing. A,B) The observed species number A) and Chao1 diversity index B) of enteric bacteria in male recipients was measured. Significant differences are indicated: Wilcoxon rank sum test. *n* = 5 per group. C,D) The observed species number C) and Chao1 diversity index D) of enteric bacteria in female recipients was measured. Significant differences are indicated: Wilcoxon rank sum test. *n* = 5 per group. E,G) PCoA were used to examined the alteration of intestinal bacteria taxonomic pattern from male E) and female G) recipients. *n* = 5 per group. F,H) The β diversity of enteric bacteria was compared by weighted unifrac analysis. Significant differences are indicated: Wilcoxon rank sum test. *n* = 5 per group. I,J) Kaplan–Meier analysis I) and body weight measurement J) of male recipients after 7 Gy TBI, *n* = 18 per group; *P < 0.05, **P < 0.01 by Student's *t*‐test between simvastatin‐treated group and control. K,L) Kaplan–Meier analysis K) and body weight measurement L) of female recipients after 7 Gy TBI, *n* = 18 per group.

We performed fecal microbiota transplantation using gut microbes from opposite sexual donors to rebuild the sex‐characteristic gut microbiota structure and further elucidate the underlying mechanism of simvastatin and HFD mitigating irradiation‐caused toxicity in a sex‐dependent fashion (Figure S16A, Supporting Information). After 14 d of FMT, we assessed the intestinal microbiota structure of recipients. Male and female recipients receiving opposite sexual enteric microbes harbored higher abundance of intestinal bacteria (**Figure** **9**A–D; Figure S16B, C, Supporting Information). As expected, FMT also shifted the intestinal bacterial flora profile of male and female recipients (Figure 9E–H; Figure S17A–D, Supporting Information), indicating that FMT indeed shapes the enteric bacterial composition pattern of male and female recipients. Next, the recipients were treated with simvastatin or HFD following 7 Gy TBI. Intriguingly, simvastatin failed to protect male recipients against radiation‐induced toxicity, covering unchanged survival rate and incremental weight loss (Figure 9I,J). For female recipients, HFD led to no significant alterations of survival rate and lower body weight (Figure 9K,L), suggesting that the curative effects of simvastatin and HFD depend on the sex‐distinct gut microbiota pattern. Together, the data suggests that simvastatin and HFD lose therapeutic efficacy toward radiation‐associated injury for male and female mice with intestinal flora imbalance.

## 3 Discussion

Radiotherapy is routinely used for localized cancer or isolated metastasis, and as a palliative manipulation in patients with a wide range of diseases, approximately 50–60% of cancer diagnosis has an indication for radiotherapy during the therapeutic course and 40% of patients are cured.^33^, ^34^ The delivery of ionizing radiation in any form promotes intracellular production of reactive oxygen species and release of endogenous danger signals, precipitating inflammatory damage in adjacent nonmalignant tissues.^1^ Clinically, adverse side effects of radiotherapy range from fatigue, nausea, hair loss, skin irritation, anemia, infertility, cardiovascular disease, cognitive impairment, and even to the development of secondary cancers. Take abdominal and pelvic cancer for instance, radiotherapy is intertwined with gastrointestinal syndromes, such as malabsorption, bacterial enteritis, and diarrhea, which hinders treatment progression and even leads to death.^35^, ^36^ Driven by a deeper understanding of cancer biology, improved surgical outcomes and increasingly efficacious multimodal chemotherapy and radiotherapy regimens, a mounting number of cancer patients can survive the aliment.^37^ Thus, preventing or reducing acute and chronic complications enmeshed with radiotherapy has increasingly become a priority.^7^ Our previous study proved that FMT was able to fight against radiation‐associated toxicity.^18^ However, in clinical settings, application of FMT is limited by aesthetic concerns, costs of donor screening, material preparation as well as potential pitfalls.^38^, ^39^ Overall, radiotherapy‐associated adverse side effects remain a conundrum required effective treatments. And in this study, we are more concerned about how to mitigate radiotherapy‐induced GI toxicity of patients with pelvic and abdominal malignancies. Previously, we observed that FMT mitigated radiation‐caused injury through reprogramed the signaling pathways involved in metabolism.^18^ Thus, male and female mice were exposed to irradiation and fed with HFD, in overt opposition to male mice, we found that female animals showed a series of improved signs for the first time. We further used simvastatin, atorvastatin, and rosuvastatin to block lipid metabolism, only simvastatin protected male mice against radiation‐induced toxicity. Of note, simvastatin had been reported to mitigate radiation‐induced organ injuries, covering small intestine and hematopoiesis system.^26^, ^40^ However, the mice in these studies were exposed to total body irradiation only which caused acute hematopoietic syndrome not GI syndrome. Importantly, the effects of simvastatin on irradiated female mice were absolutely missed, limiting the usage of simvastatin in patients after radiation therapy. In our present study, we obtained that oral gavage of simvastatin further lessened the weight of hematogenic organs in irradiated female mice, resulting in deterioration of injury mediated by radiation exposure. Collectively, unwanted nuclear exposure drives sophisticated injury inextricably; however, the responses of male and female victims to special treatment are quite diverse. In clinical application, our findings support that the rehabilitation course after radiotherapy to pelvic and abdominal malignancies should base on the gender of patients. In a word, simvastatin is good for male patients, and HFD is good for female patients.

The difference between male and female in features, such as body size, brain anatomy, and immune responses, is termed as sexual dimorphism. Sexual dimorphism is ubiquitous in animals and facilitates a wide range of morphological, physiological, and functional parameters. In the case of short‐term fasting, liver metabolic strategies show a major sexual dimorphism in the use of amino acid as source of fuel for the production of lipids.^41^ Epidemiological evidence points to sexual dimorphism as a relevant element for cancer incidence and survival, empirically, the incidence of types of cancer arising in organs with nonreproductive functions is higher in male populations than in female populations, with associated differences in survival.^42^ However, most preclinical and clinical medical research has been biased with respect to sex. There has been a tendency to treat the male and female patients as equivalent and not consider how fluctuations of sexual difference in experimental settings. The failure to include both sexes in experiments, together with insufficient analysis of data by sex, in fact generates data not biologically relevant to either sex.^43^ The National Institutes of Health (NIH) has recently mandated researchers to consider sex as a biological variable in preclinical research, by including both sexes in research designs.^25^ The inability to have a clear vision of the involvement of sex in a pathological and pharmacological manifestation limits our insights into the mechanism inducing the disease and our ability to establish effective prevention and curation plans. Cancer patients receive pelvic or abdominal radiotherapy with a 60–80% incidence of acute radiation enteropathy. Clinically, free‐radical scavenger amifostine is the only drug currently approved by the FDA for reduction of radiation therapy adverse side effects for both male and female patients.^44^ However, our previous and the present studies underpin that although the clinical adverse side effects associated with irradiation are similar, the molecular responses of male and female animals toward irradiation are diverse, such as the shifts of gut microbiota and the alterations of gene expression profile of special organs.^18^ It is implying that traditional treatment for male and female patients might represent different effects. As expected, we fed irradiated male and female with HFD and obtained that HFD overtly mitigated the radiation‐induced toxicity of female mice but not male mice. Contrarily, oral gavage of simvastatin alleviated radiation‐caused hematopoietic system and GI tract injury, further supporting that simvastatin is an efficacious therapeutic agent for male patients but HFD for female patients after radiation therapy in preclinical settings. Long‐term HFD, such as a period of 9 to 14 months feeding, has been reported to drive stemness and tumorigenicity of intestinal progenitors.^45^ Here, the irradiated female mice were fed with HFD for only 15 d which was a quite short duration compared with 9 month. However, whether short‐term HFD eliciting tumorigenesis in irradiated female experimental models still require further study. Simvastatin inhibits HMGCR, thus, analysis the survival rate of cancer patients based on expression of HMGCR could imply the effects of simvastatin for the prognosis of the patients. On the basis of bioinformatics analysis, we obtained that prostatic cancer patients with low level of HMGCR and ovarian cancer patients with high level of HMGCR meant better prognosis.

Flora disequilibrium of enteric microbial members impact substantial aspects of host biology, such as cardiovascular and metabolic diseases as well as development of certain cancers.^46^, ^47^ Our previous study has reported that the intestinal bacteria taxonomic proportions of male and female mice are different.^18^ Depletion of gut microbes by antibiotic treatment triggers sexually biased microglial responses of adult mice,^48^ suggesting that sexual distinct gut microbiota might relate to sexual dimorphism. Accordingly, we performed FMT to restructure sex‐specific intestinal bacterial flora profile of recipients using gut microbes from opposite sexual donors, and aimed to explore the mechanism by which simvastatin and HFD protect against radiation injuries in a sex‐dependent manner. After 14 d of FMT, the recipients received simvastatin or HFD following radiation exposure. Intriguingly, oral gavage of simvastatin or feeding with HFD unchanged the survival rate and body weight of irradiated male or female recipients. Importantly, simvastatin administration or HFD mitigated the radiation‐induced toxicity of female or male recipients respectively, indicating that sex‐specific enteric microbes underpin the curative effects of simvastatin or HFD for male or female. Regardless of drug administration by enteral or parenteral route, gut microbiota impacts drug pharmacokinetics, activity, and toxicity at various levels, with three main clinical outcomes: facilitation of drug efficacy; abrogation and compromise of pesticide effects; and mediation of toxicity.^5^, ^49^, ^50^ Different bacterial species increase the response to one drug yet decrease the effect of another.^51^ Specifically, it has been reported that gut microbes orchestrate the effects of fluoropyrimidines through metabolic drug interconversion involving bacterial vitamin and ribonucleotide metabolism.^52^ In this regard, gut microbiota might be a key element governing the therapeutic efficiency toward radiotherapy‐enmeshed adverse side effects. However, many nonantibiotics drugs are able to educate and tune the enteric microbiota structure of recipients.^53^ Here, simvastatin and HFD administration shaped the intestinal bacteria taxonomic proportions of irradiated male and female mice. Importantly, ABX‐challenged, microbiota‐depleted mice unresponsed to simvastatin or HDF treatment following irradiation, representing as serious hematopoietic and GI toxicity, suggesting that simvastatin or HDF interacts with intestinal microorganisms to perform radioprotective functions for male or female. In addition, transfer of gut microbes from adult males to immature females shaped the recipient's microbiota, resulting in elevated testosterone and metabolomic fluctuations, reduced islet inflammation and autoantibody production, and robust T1D protection,^54^ suggesting that sex bias in gut microbiome modulates autoimmunity of host. We have proved that fecal microbiota transplantation can be employed to fight against radiation injuries. In our opinion, this remedy is completely different from administration of simvastatin and HFD. In the first place, performance of FMT replaces the imbalanced gut flora and modulates the intestinal microecology. However, the therapeutical effects of simvastatin and HFD to radiation toxicity depend on sex‐distinct gut microbiota structure, which in turn regulate enteric bacterial taxonomic pattern. In the next place, the gene expression profiles of recipients after FMT or simvastatin (and HFD) are quite diverse. For instance, simvastatin and HFD administration elicits rhythm‐related signaling response in small intestine tissues from irradiated mice, which is not main alteration after FMT. In addition, *Escherichia coli* has been reported to serve as a key driver in colorectal cancer development since the bacterium causes chronic inflammation during inflammatory bowel disease predisposing an individual cancerous transformation.^55^, ^56^ In the present study, simvastatin treatment elevated the frequency of *Escherichia coli* in irradiated female mice which might be the reason for oral gavage of simvastatin elicits systemic inflammation of female mice after irradiation (Figure 5K). Gut commensal *Bacteroides acidifaciens* has been proved to prevent obesity and enhance insulin sensitivity,^57^ and the frequency of *Bacteroides acidifaciens* is negative correlation with inflammation stage.^58^ In line with the previous studies, irradiated male mice represented lower systemic inflammation stage with *s_Bacteroides_acidifaciens* as a dominant strain in intestine (Figure S10C, Supporting Information). Together, owing to the sex different in intestinal microbiota pattern, the chosen optimal therapeutic strategies to fight against radiotherapy‐associated adverse side effects should based on the gender of patients.

## 4 Conclusions

Here, our present study demonstrates that irradiated male and female received a special therapeutic option represent diverse responses. In detail, oral gavage of simvastatin ameliorates hematopoietic system injury, improves GI tract function and epithelial integrity only in irradiated males, but feeding with HFD overtly mitigates bone marrow and GI toxicity merely in irradiated females. Notably, antibiotics challenging or transplantation of gut microbiota from opposite sexual donors eradicates the curative effect of simvastatin or HFD in respective genders. Mechanistically, simvastatin and HFD retained the miRNA expression profile, reprogramed the spectrum of mRNA expression in small intestine tissues and enteric bacterial taxonomic pattern from TAI‐exposed male and female mice. Our observations now suggest that rehabilitation strategies for cancer patients receiving radiotherapy should take the gender of patients into account. To improve prognosis, male patients undergoing radiotherapy should be given simvastatin, but not high fat diet, to fight against radiation‐associated side effects; whereas female patients could be given high fat diet, but not simvastatin, during radiation therapy.

## 5 Experimental Section

Information regarding the following aspects of this study is available in the Supporting Information: mice, irradiation study, drug and high fat diet administration, reverse transcription polymerase chain reaction (RT‐PCR), measurement of spleen, thymus gland, and colon, histology, FITC–dextran permeability experiments, quantification of IL‐6, TNFɑ and LCN2 by ELISA, measurement of malondialdehyde, donor stool preparation and administration, bacterial diversity analysis, RNA quantification and qualification for sequencing, library preparation for transcriptome sequencing, library preparation for small RNA sequencing, KEGG enrichment analysis of differentially expressed genes, antibiotics test, and statistical analysis. Animal experiments were performed according to the institutional guidelines approved by the Animal Care and Ethics Committee of IRM‐PUMC, which complied with the Guide for the Care and Use of Laboratory Animals and the National Institutes of Health guide for the Care and Use of Laboratory Animals.

## Conflict of Interest

The authors declare no conflict of interest.

## Supporting information

Supplementary
