# Supplementary material for: Sexual Dimorphism of Gut Microbiota Dictates Therapeutics Efficacy of Radiation Injuries
Source: Adv Sci (Weinh). 2019 Aug 29;6(21):1901048. doi: 10.1002/advs.201901048 (PMC6839645; doi:10.1002/advs.201901048)
Supplement: Supplementary file 1 — Supplementary [file ADVS-6-1901048-s001.pdf]

Copyright WILEY-VCH Verlag GmbH & Co. KGaA, 69469 Weinheim, Germany, 2019.

ADVANCED SCIENCE
Open Access

Supporting Information

for *Adv. Sci.,* DOI: 10.1002/advs.201901048

Sexual Dimorphism of Gut Microbiota Dictates Therapeutics Efficacy of Radiation Injuries

Ming Cui, Huiwen Xiao, Yuan Li, Shuqin Zhang, Jiali Dong, Bin Wang, Changchun Zhu, Mian Jiang, Tong Zhu, Junbo He, Haichao Wang, and Saijun Fan**

Supplementary information

Materials and methods

Mice

Six to eight-week-old male and female C57BL/6J mice were purchased from Beijing Huafukang Bioscience Co. Inc (Beijing, China). Mice were housed in the Specific Pathogen Free level animal facility at the Institute of Radiation Medicine (IRM), the Chinese Academy of Medical Sciences (CAMS). Mice were kept under standard conditions (ambient temperature 22±2℃, air humidity 40-70% and a 12/12-h light/dark cycle) and continuous access to a standard diet and water. All male and female mice in this study were of a pure C57BL/6 genetic background and separated into groups randomly. Animal experiments were performed according to the institutional guidelines approved by the Animal Care and Ethics Committee of IRM-PUMC, which complied with the Guide for the Care and Use of Laboratory Animals and the National Institutes of Health guide for the Care and Use of Laboratory Animals.

Irradiation study

A Gammacell-40 137Cs irradiator (Atomic Energy of Canada Limited, Chalk River, ON, Canada) at a dose rate of 0.88 Gy per minute was used for all experiments. Male (approximately 20 g in body weight) and female (approximately 18 g) mice treated with total body irradiation (TBI) were exposed to γ-ray (4 Gy or 7 Gy) or total abdominal irradiation (TAI) were exposed to 12 Gy. Control mice were sham-irradiated. The weight of the mice in this study was assessed for 5 days.

Drug and high fat diet administration

Simvastatin tablets (Zocor, Merck Sharp & Dohme Limited, Hoddesdon, Herts, UK) administration *via* oral route were performed for 15 days consecutively. Simvastatin was once daily administered at a dose of 4 mg/kg/day (at 2 p.m.) and was firstly administered at the day of irradiation. High fat diet (DIO series Diets, H10045, 20% kcal from protein, 35% kcal from carbohydrate and 45% kcal from fat, Beijing Huafukang Bioscience Co. Inc, Beijing, China) was began at the day of irradiation and fed for 15 days consecutively.

Antibiotics test

The male and female mice in simvastatin or HFD groups were housed with Ciprofloxacin (125mg/L, Sigma-Aldrich, Madrid, Spain), Metronidazole (100mg/L, Sigma-Aldrich, Madrid, Spain), Vancomycin (50mg/L, Sigma-Aldrich, Madrid, Spain), Streptomycin (100U/L, Solarbio, Beijing, China) and Penicillin (100U/L, Solarbio, Beijing, China) in their drinking water for 20 days before irradiation and kept drinking thoughout the whole experiment. The fresh antibiotic solution was prepared every day to promise its activity. The mice in control and TAI groups were without antibiotics treatment.

Reverse transcription polymerase chain reaction (RT-PCR)

Total RNA was extracted from tissues using TRIZOL reagent (Invitrogen, Carlsbad, CA, USA). Reverse transcription was performed with PrimeScript RT Reagent Kit (Perfect Real Time) (TaKaRa Biotechnology, Dalian, China). For mature microRNA detection, total RNA was polyadenylated by poly(A) polymerase (Ambion, Austin, TX, USA) as described previously. [1] cDNA was produced by using poly(A)-tailed total RNA and reverse transcription primer with ImProm-IIReverse Transcriptase (Promega, Madison, WI, USA), according to the manufacturer's instructions. The primers are listed in Supplementary Table S1. GAPDH (glyceraldehyde 3-phosphate dehydrogenase) and U6 were used as control for protein coding genes and microRNA, respectively.

Measurement of spleen, thymus gland and colon

Male and female C57BL/6J mice were treated with single dose of 4 Gy. After 21 days, the mice were euthanized, and the spleens and thymus were excised and measured. For colon measurement, the male and female mice were exposed to 12 Gy total abdominal irradiation. The mice were euthanized, and colons were excised and measured after 21 days.

Histology

Following euthanasia, the small intestines of mice were fixed in 4% buffered formalin overnight at room temperature and then embedded in paraffin. Tissues were sectioned at 5 μm

thickness and dipped in hematoxylin and eosin (H&E) using standard protocols. For PAS staining, the small intestines of mice were fixed in Carnoy's Fluid. Dewaxed sections were hydrated and incubated in 1% periodic acid for 10 min followed by incubation in Schiff's reagent for 10 min. Sections were counterstained with Mayer's hematoxylin for 30 s, washed and dehydrated before mounting with Pertex.

FITC–dextran permeability experiments

Male and female mice administrate with simvastatin, HFD or sterile water following 12 Gy TAI, sham-irradiated mice were used as control. After 21 days, mice were fasted 14 h and gavaged with 60 mg per 100 g body weight of fluorescein isothiocyanate–dextran (FITC–dextran, FD4, 3000–5000 kD, Sigma-Aldrich, Madrid, Spain) in a volume of 0.2 ml. Blood samples were obtained by cardiac puncture at 4 h after administration of FITC–dextran and centrifuged at 860 g for 5 min at room temperature to obtain the serum. Fluorescence intensity of each serum sample (DTX 880 Multimode Detector, Beckman Coulter, CA, USA) was measured.

Quantification of IL-6, TNFα and LCN2 by ELISA

For IL-6 and TNFα measurement, plasma was collected using EDTA as an anticoagulant. Centrifuge for 15 min at 1000g, 4℃. Small intestine tissues were ground in saline. Sample dilutions of 1:4 were used for the determination of IL-6 and TNFα levels using ELISA kit (CUSABIO, MD, USA) according to the manufacturer's protocol. For LCN2 measurement, frozen faecal samples were re-suspended in PBS containing 0.1% Tween 20 to a final concentration of 0.1g/ml, then vortexed to produce a homogenous faecal suspension, followed by centrifuging for 10 min at 14,000g and 4°C. LCN2 level was measured from the clear supernatant using Mouse Lipocalin (LCN2) ELISA kit (Solarbio, Beijing, China) according to the manufacturer's instructions. Optical density was read at 450nm (Rayto, Shenzhen, China).

Measurement of malondialdehyde

The levels of malondialdehyde (MDA) in peripheral blood and small intestine were assessed using a detection kit from Solarbio (Solarbio, Beijing, China) according to the manufacturer's

instructions. Levels of MDA were evaluated and calculated by the following formula, according to the manufacturer's instructions. The levels of MDA (nmol) in the small intestine: = 25.8 × (A532 – A600).

Donor stool preparation and administration

FMT was performed based on the previous study. [2] Briefly, the healthy 6- to 8-week-old male and female C57BL/6J mice were kept in same housing and environmental conditions. Donor stool was freshly prepared on the day of transplant and that in all cases was prepared and transplanted within 4 h. Donor stool was weighed and diluted with 1 ml of saline per 0.1 g of stool. The stool was steeped in saline for about 15 min, shaken and then centrifuged at 800 rpm for 3 min. The supernatant was obtained for treatment. After FMT, male (approximately 22 g) and female (approximately 20 g) mice were treated with single dose of 7 Gy at a rate of 0.88 Gy/min.

Bacterial diversity analysis

Stool samples were freshly collected and stored at -80°C until use. DNA was extracted from the stool using the Power Fecal® DNA Isolation Kit (MoBio Carlsbad, CA USA). The DNA was recovered with 30ml of buffer in the kit. PCR products were mixed in equidensity ratios. Then, mixture PCR products were purified with Qiagen Gel Extraction Kit (Qiagen, Germany). The 16S ribosomal RNA (rRNA) V4 gene was analyzed to evaluate the bacterial diversity using Illumina HiSeq (Novogene Bioinformatics Technology Co., Ltd.). Sequences analysis was performed by Uparse software (Uparse v7.0.1001, http://drive5.com/uparse/). Sequences with ≥97% similarity were assigned to the same OTUs. Representative sequence for each OTU was screened for further annotation. For each representative sequence, the Silva123 Database was used based on RDP classifier (Version 2.2, http://sourceforge.net/projects/rdpclassifier/) algorithmto annotate taxonomic information. For gut microbiota analysis, we collected faecal pellets from different cages to avoid cage effects. The primers are listed in Supplementary Table 1.

RNA quantification and qualification for sequencing

RNA degradation and contamination was monitored on 1% agarose gels. RNA purity was checked using the NanoPhotometer® spectrophotometer (IMPLEN, CA, USA). RNA concentration was measured using Qubit® RNA Assay Kit in Qubit®2.0 Flurometer (Life Technologies, CA, USA). RNA integrity was assessed using the RNA Nano 6000 Assay Kit of the Bioanalyzer 2100 system (Agilent Technologies, CA, USA).

Library preparation for transcriptome sequencing

A total amount of 3 μg RNA per sample was used as input material for the RNA sample preparations. Sequencing libraries were generated using NEBNext® UltraTM RNA Library Prep Kit for Illumina® (NEB, USA) following manufacturer's recommendations and index codes were added to attribute sequences to each sample. Briefly, mRNA was purified from total RNA using poly-T oligo-attached magnetic beads. Fragmentation was carried out using divalent cations under elevated temperature in NEBNext First Strand Synthesis Reaction Buffer (5X). First strand cDNA was synthesized using random hexamer primer and M-MuLV Reverse Transcriptase (RNase H^-). Second strand cDNA synthesis was subsequently performed using DNA Polymerase I and RNase H. Remaining overhangs were converted into blunt ends via exonuclease/polymerase activities. After adenylation of 3′ ends of DNA fragments, NEBNext Adaptor with hairpin loop structure were ligated to prepare for hybridization. In order to select cDNA fragments of preferentially 250~300 bp in length, the library fragments were purified with AMPure XP system (Beckman Coulter, Beverly, USA). Then 3 µl USER Enzyme (NEB, USA) was used with size-selected, adaptor-ligated cDNA at 37°C for 15 min followed by 5 min at 95 °C before PCR. Then PCR was performed with Phusion High-Fidelity DNA polymerase, Universal PCR primers and Index (X) Primer. At last, PCR products were purified (AMPure XP system) and library quality was assessed on the Agilent Bioanalyzer 2100 system.

Library preparation for small RNA sequencing

A total amount of 3 µg total RNA per sample was used as input material for the small RNA library. Sequencing libraries were generated using NEBNext® Multiplex Small RNA Library Prep Set for Illumina® (NEB, USA.) following manufacturer's recommendations and index

codes were added to attribute sequences to each sample. Briefly, NEB 3' SR Adaptor was directly, and specifically ligated to 3' end of miRNA, siRNA and piRNA. After the 3' ligation reaction, the SR RT Primer hybridized to the excess of 3′ SR Adaptor (that remained free after the 3′ ligation reaction) and transformed the single-stranded DNA adaptor into a double-stranded DNA molecule. This step is important to prevent adaptor-dimer formation, besides, dsDNAs are not substrates for ligation mediated by T4 RNA Ligase 1 and therefore do not ligate to the 5′SR Adaptor in the subsequent ligation step. 5′ ends adapter was ligated to 5′ ends of miRNAs, siRNA and piRNA. Then first strand cDNA was synthesized using M-MuLV Reverse Transcriptase (RNase H^{-}). PCR amplification was performed using LongAmp Taq 2X Master Mix, SR Primer for illumina and index (X) primer. PCR products were purified on an 8% polyacrylamide gel (100V, 80 min). DNA fragments corresponding to 140~160 bp (the length of small noncoding RNA plus the 3′ and 5′ adaptors) were recovered and dissolved in 8 μL elution buffer. At last, library quality was assessed on the Agilent Bioanalyzer 2100 system using DNA High Sensitivity Chips.

KEGG enrichment analysis of differentially expressed genes

KEGG is a database resource for understanding high-level functions and utilities of the biological system, such as the cell, the organism and the ecosystem, from molecular-level information, especially large-scale molecular datasets generated by genome sequencing and other high-through put experimental technologies (http://www.genome.jp/kegg/). We used cluster Profiler R package to test the statistical enrichment of differential expression genes in KEGG pathways.

Statistical analysis

The data are presented as the means ± SEM with respect to the number of samples (n) in each group. Significance was assessed by comparing the mean values (6 standard deviation; SD) using Student’s *t*-test and Wilcoxon rank sum test for independent groups as follows: * $P < 0.05$, ** $P < 0.01$, *** $P < 0.005$. Kaplan-Meier analysis was performed for survival analysis of mouse models, and significance between survival curves was determined by a log rank test. Kaplan-Meier analysis for prostatic cancer and ovarian cancer patients was performed based

on http://gepia.cancer-pku.cn/ and http://kmplot.com/analysis/ individually. Results with P < 0.05 were considered statistically significant.

Reference

[1] M. Cui, Z. L. Xiao, Y. Wang, M. Y. Zheng, T. Q. Song, X. L. Cai, B. D. Sun, L. H. Ye, X. D. Zhang, Cancer Res. **2015**, 75, 846.

[2] M. Cui, H.W. Xiao, Y. Li, L.X. Zhou, S.Y. Zhao, D. Luo, Q.S. Zheng, J.L. Dong, Y. Zhao, X. Zhang, J.L. Zhang, L. Lu, H.C. Wang, S.J. Fan, EMBO Mol. Med. **2017**, 9, 448.

Supplementary figures and figure legends

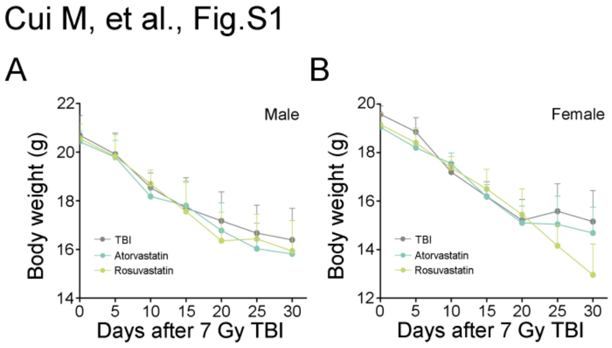

Figure S1. Atorvastatin and rosuvastatin unchange the body weight of male or female mice after irradiation exposure. A, B) Body weight was compared among three group of male (A) and female (B) mice after 7 Gy TBI, Significant differences are indicated by Student's *t*-test between each two cohort, n=24 per group.

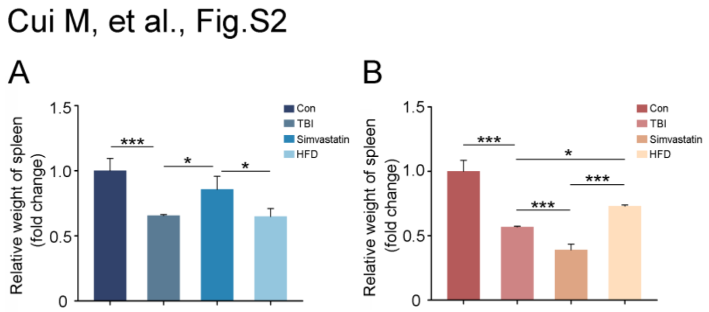

Figure S2. Statistical analysis of the weight of spleen tissues from mice after 7 Gy TBI. A) Relative weight of spleen from male mice. B) Relative weight of spleen from female mice.

The mice without TBI are as control. Significant differences are indicated: * P < 0.05, *** P < 0.005 by Student's *t*-test between each two cohort, n = 18 per group.

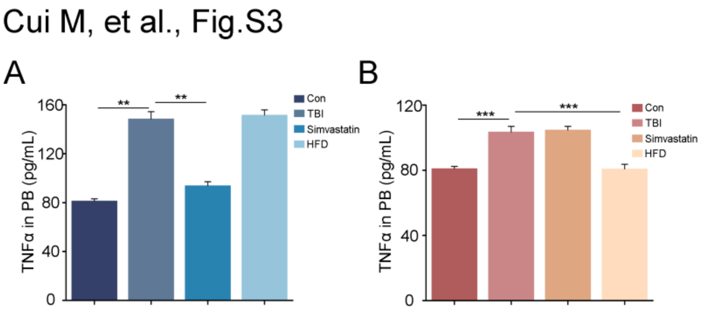

Figure S3. Simvastatin or HFD reduces the inflammatory marker in PB from male or female mice after 7 Gy TBI. A, B) The levels of TNFa in PB of male (A) and female (B) were examined by ELISA. Significant differences are indicated: ** P <0.01, *** P < 0.005 by Student's *t*-test between each two cohort, n=18 per group.

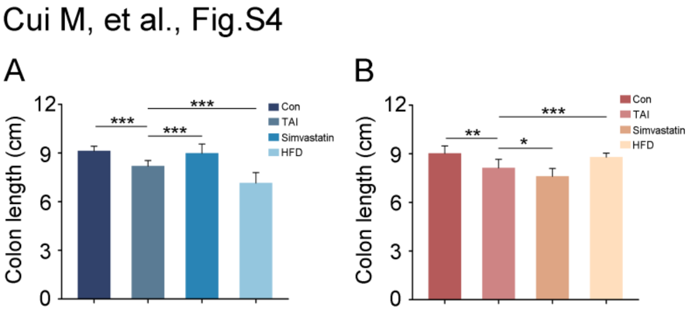

Figure S4. Statistical analysis of dissected colon. A, B) The colon length was measured from male (A) and female (B) mice after 12 Gy TAI in four cohorts. Significant differences are indicated: * P <0.05, ** P <0.01, *** P < 0.005 by Student's *t*-test between each two cohort, n=24 per group.

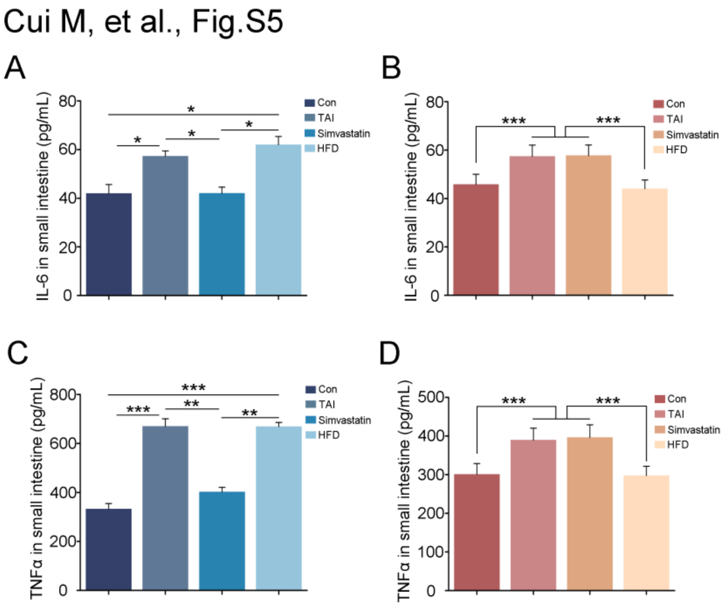

Figure S5. Simvastatin or HFD reduces the inflammatory marker in small intestine from male or female mice after 12 Gy TAI. A, B) The levels of IL-6 in small intestine tissues of male (A) and female (B) were examined by ELISA. C, D) The levels of TNFa in small intestine tissues of male (C) and female (D) were examined by ELISA. Significant differences are indicated: * P <0.05, ** P <0.01, *** P < 0.005 by Student's *t*-test between each two cohort, n=24 per group.

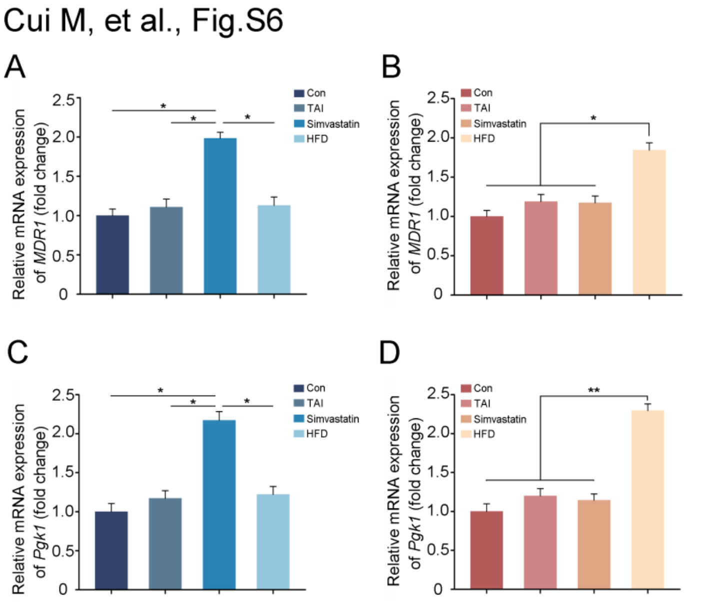

Figure S6. Simvastatin or HFD improves the integrity of small intestine from mice after 12 Gy TAI. A, B) The expression levels of *MDR1* are examined in small intestine tissues from

male (A) and female (B) mice by quantitative PCR. C, D) The expression levels of *Pgk1* are examined in small intestine tissues from male (C) and female (D) mice by quantitative PCR. Significant differences are indicated: * P <0.05, ** P <0.01 by Student's *t*-test between each two cohort, n=24 per group.

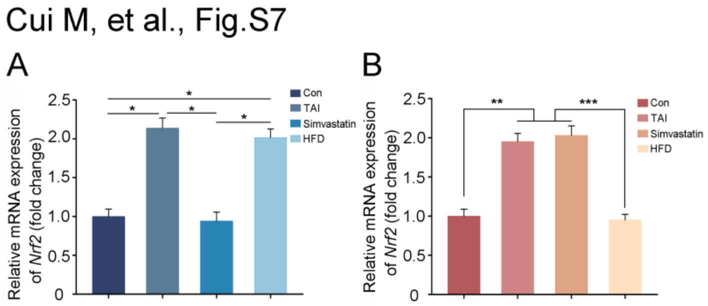

Figure S7. Simvastatin and HFD fight against radiation-elevated cytotoxic reactive oxygen specie. A, B) The expression levels of *Nrf2* are examined in small intestine tissues from male (A) and female (B) mice by quantitative PCR. Significant differences are indicated: * P <0.05, ** P <0.01, *** P < 0.005 by Student's *t*-test between each two cohort, n=24 per group.

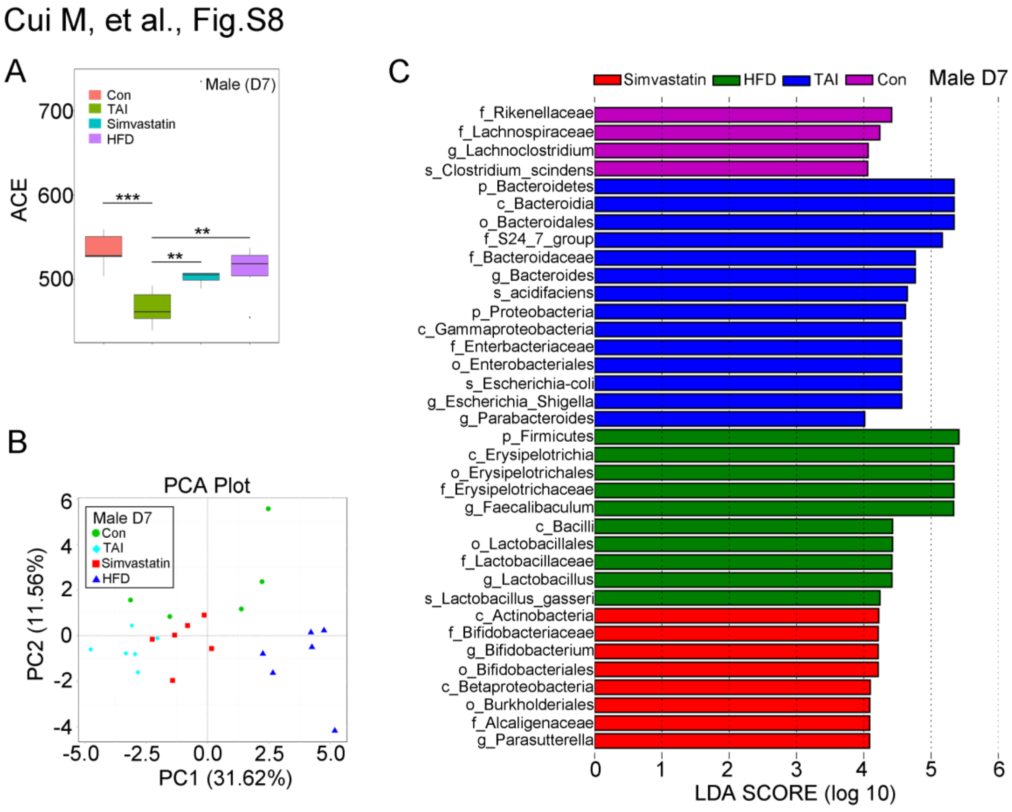

Figure S8. Oral gavage of simvastatin and HFD educate irradiation-shifted intestinal bacterial structure of male mice at day 7 after TAI. A) The ACE diversity index of intestinal bacteria

was assessed by 16S rRNA high-throughput sequencing at day 7 after TAI. Significant differences are indicated: Wilcoxon rank sum test. The top and bottom boundaries of each box indicate the 75th and 25th quartile values, respectively, and lines within each box represent the 50th quartile (median) values. Ends of whiskers mark the lowest and highest diversity values in each instance. n = 5 (control group) or 6 (irradiated groups). B) Principal component analysis (PCA) based on significant difference at the genus level was used to show the shift in intestinal bacterial composition profile after irradiation at day 7. C) Linear discriminant analysis (LDA) effect size (LEfSe) results represented significantly different in abundance of gut bacteria among the four groups and indicated the effect size of each differentially abundant bacterial taxon in the small intestine after irradiation at day 7, n = 5 (control group) or 6 (irradiated groups). Significant differences are indicated: Wilcoxon rank sum test.

Cui M, et al., Fig.S9

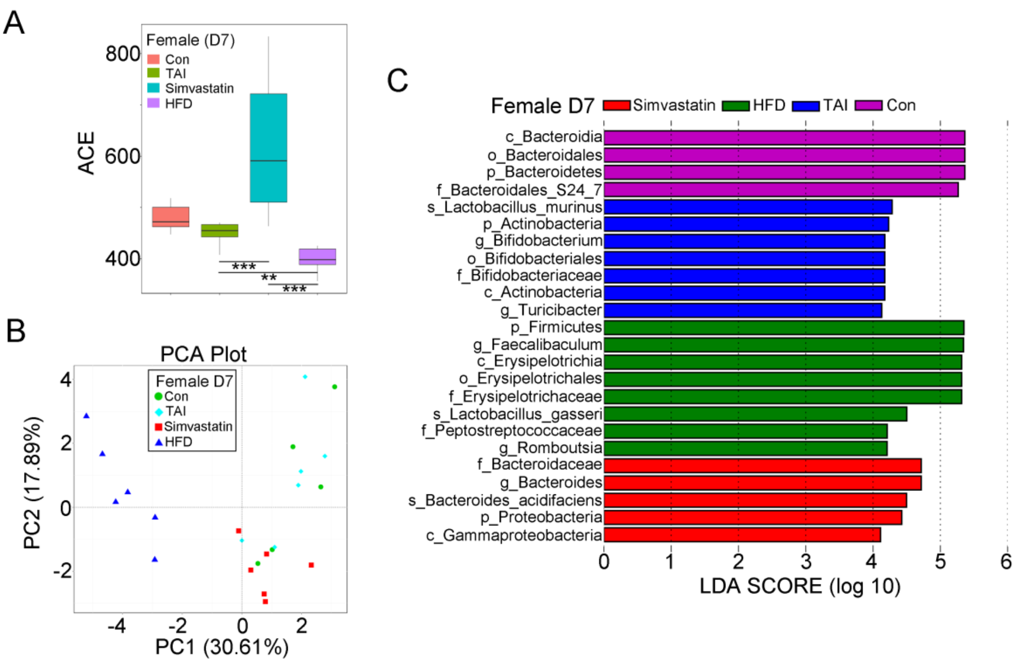

Figure S9. Oral gavage of simvastatin and HFD educate irradiation-shifted intestinal bacterial structure of female mice at day 7 after TAI. A) The ACE diversity index of intestinal bacteria was assessed by 16S rRNA high-throughput sequencing at day 7 after TAI. Significant differences are indicated: Wilcoxon rank sum test. n = 5 (control group) or 6 (irradiated groups). B) Principal component analysis (PCA) based on significant difference at the genus level was used to show the shift in intestinal bacterial composition profile after irradiation at day 7. C) Linear discriminant analysis (LDA) effect size (LEfSe) results represented

significantly different in abundance of gut bacteria among the four groups and indicated the effect size of each differentially abundant bacterial taxon in the small intestine after irradiation at day 7, n = 5 (control group) or 6 (irradiated groups). Significant differences are indicated: Wilcoxon rank sum test.

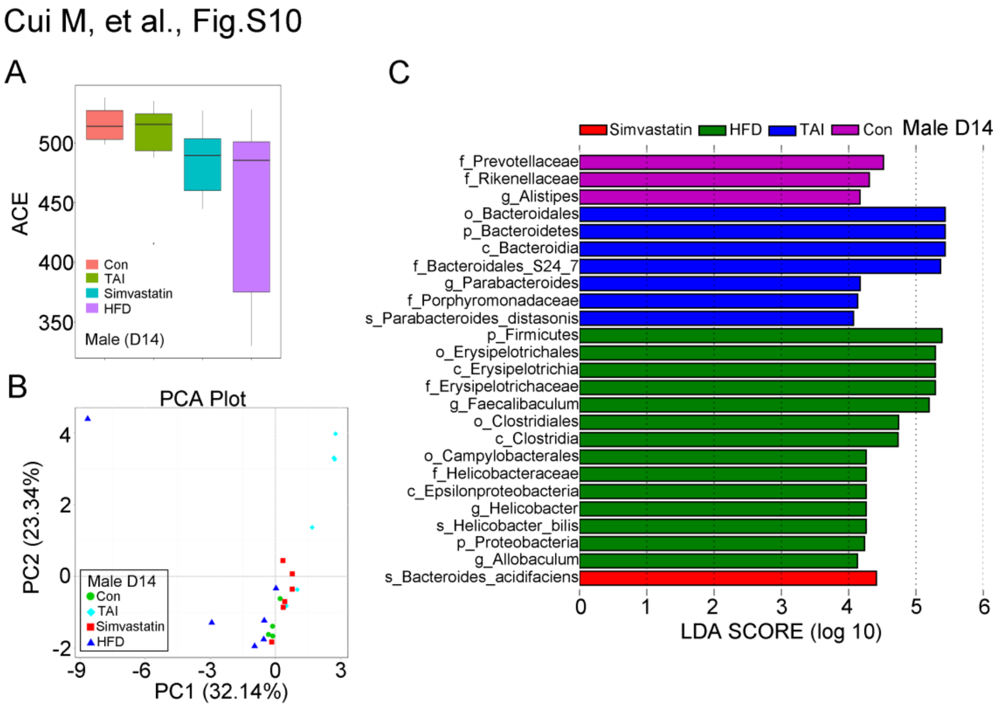

Figure S10. Simvastatin and HFD administration shape intestinal bacterial structure of male mice at day 14 after TAI. A) The ACE diversity index of intestinal bacteria was assessed by 16S rRNA high-throughput sequencing at day14 after TAI. Significant differences are indicated: Wilcoxon rank sum test. n = 5 (control group) or 6 (irradiated groups). B) Principal component analysis (PCA) based on significant difference at the genus level was used to show the shift in intestinal bacterial composition profile after irradiation at day 14. C) Linear discriminant analysis (LDA) effect size (LEfSe) results represented significantly different in abundance of gut bacteria among the four groups and indicated the effect size of each differentially abundant bacterial taxon in the small intestine after irradiation at day 14, n = 5 (control group) or 6 (irradiated groups). Significant differences are indicated: Wilcoxon rank sum test.

Cui M, et al., Fig.S11

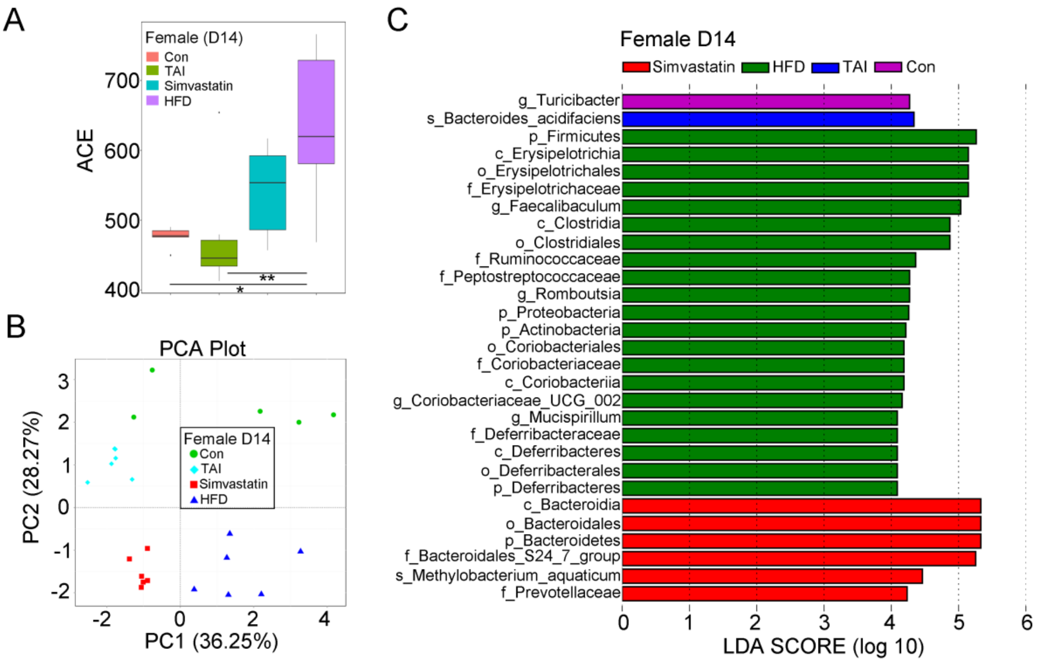

Figure S11. Simvastatin and HFD administration shape intestinal bacterial structure of female mice at day 14 after TAI. A) The ACE diversity index of intestinal bacteria was assessed by 16S rRNA high-throughput sequencing at day 14 after TAI. Significant differences are indicated: Wilcoxon rank sum test. n = 5 (control group) or 6 (irradiated groups). B) Principal component analysis (PCA) based on significant difference at the genus level was used to show the shift in intestinal bacterial composition profile after irradiation at day 14. C) Linear discriminant analysis (LDA) effect size (LEfSe) results represented significantly different in abundance of gut bacteria among the four groups and indicated the effect size of each differentially abundant bacterial taxon in the small intestine after irradiation at day 14, n = 5 (control group) or 6 (irradiated groups). Significant differences are indicated: Wilcoxon rank sum test.

Cui M, et al., Fig.S12

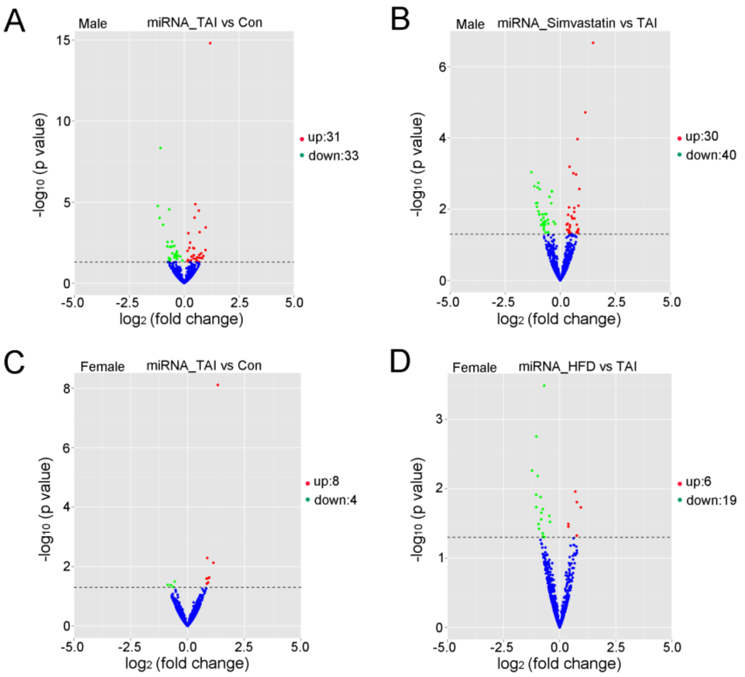

Figure S12. The optimal therapeutic options reprogram irradiation-shaped miRNA expression profile of mice small intestine. A-D) Volcano plots of identified different miRNAs from small intestine of male (A and B) and female (C and D) mice. A: control group versus TAI group, B: simvastatin group versus TAI group, C: control group versus TAI group, D: HFD group versus TAI group. In the volcano plots, each point represented a miRNA.

Cui M, et al., Fig.S13

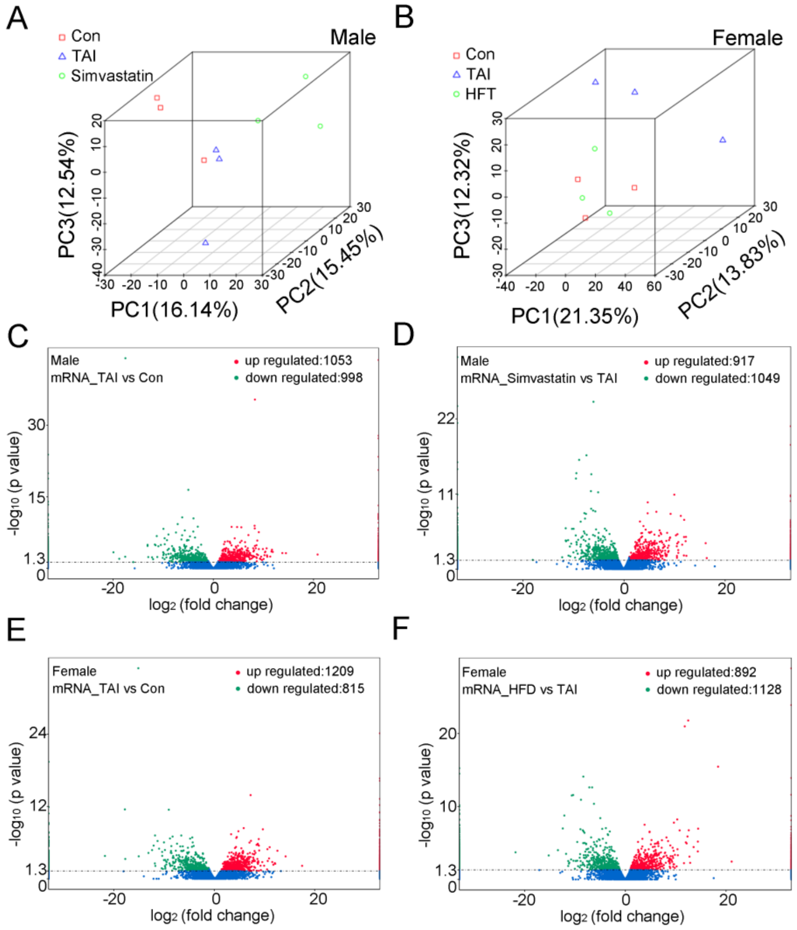

Figure S13. The optimal therapeutic options reprogram the spectrum of mRNA in small intestin tissues shaped by irradiation. A, B) PCA was used to measure the alterations of mRNA expression profile of male (A) and female (B) mice. C-F) Volcano plots of identified different mRNAs from small intestine of male (C and D) and female (E and F) mice. C: control group versus TAI group, D: simvastatin group versus TAI group, E: control group versus TAI group, F: HFD group versus TAI group. In the volcano plots, each point represented an mRNA.

Cui M, et al., Fig.S14

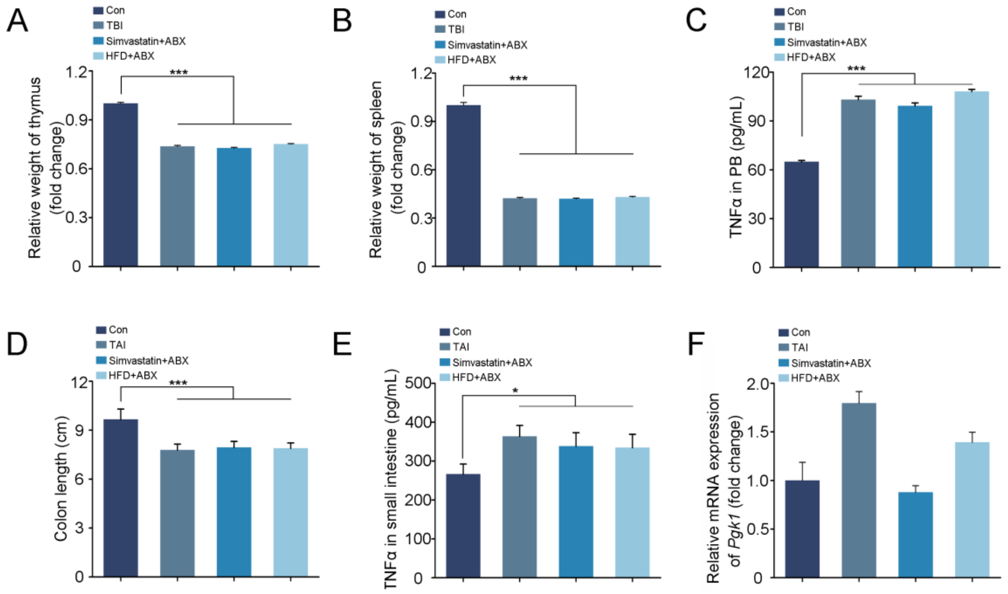

Figure S14. Gut microbiota contributes to the radioprotective effects of simvastatin and HFD in male mice. The mice were housed with antibiotic mixture (ABX) in drinking water. A-B) Weights of dissected thymuses (A) and spleens (B) from male mice in the four groups, the thymuses and spleens were obtained at day 21 after 4 Gy TBI. Significant differences are indicated: *** $P < 0.005$ by Student's *t*-test between each two cohort, n = 18 per group. C) The level of TNFα in PB was examined by ELISA. Significant differences are indicated: *** $P < 0.005$ by Student's *t*-test, n=18 per group. D) The colon length was measured in four cohorts at day 21 after 12 Gy TAI. Significant differences are indicated: *** $P < 0.005$ by Student's *t*-test between each two cohort, n=18 per group. E) The level of TNFα in small intestines was examined by ELISA. Significant differences are indicated: * $P < 0.05$ by Student's *t*-test between each two cohort, n=18 per group. F) The expression level of *Pgk1* was examined in small intestine tissues by quantitative PCR. Significant differences are indicated by Student's *t*-test between each two cohort, n=18 per group.

Cui M, et al., Fig.S15

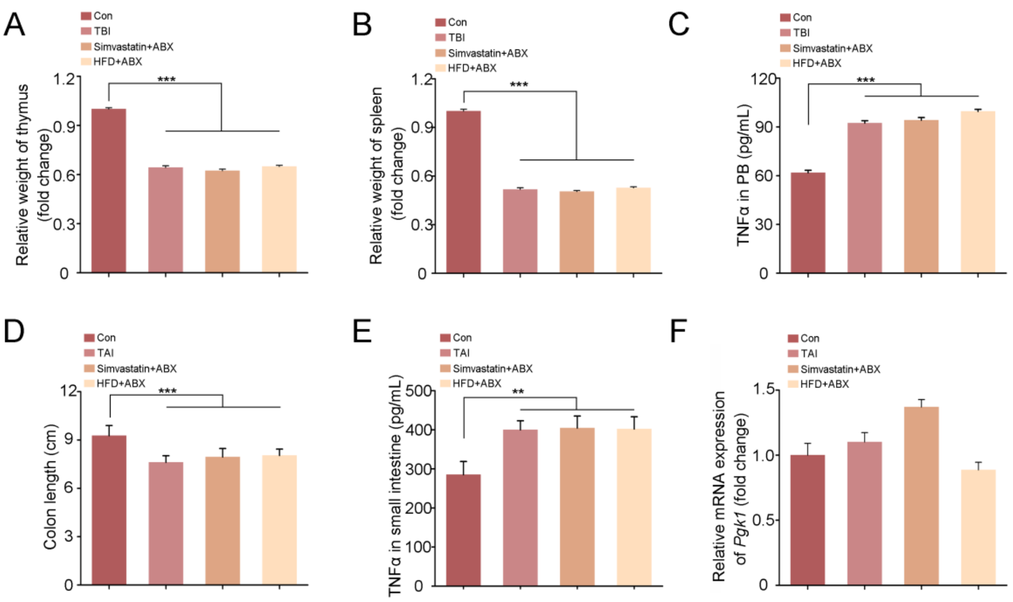

Figure S15. Gut microbiota contributes to the radioprotective effects of simvastatin and HFD in female mice. The mice were housed with antibiotic mixture (ABX) in drinking water. A-B) Weights of dissected thymuses (A) and spleens (B) from female mice in the four groups, the thymuses and spleens were obtained at day 21 after 4 Gy TBI. Significant differences are indicated: *** P < 0.005 by Student's *t*-test between each two cohort, n = 18 per group. C) The level of TNFα in PB was examined by ELISA. Significant differences are indicated: *** P < 0.005 by Student's *t*-test, n=18 per group. D) The colon length was measured in four cohorts at day 21 after 12 Gy TAI. Significant differences are indicated: *** P < 0.005 by Student's *t*-test between each two cohort, n=18 per group. E) The level of TNFα in small intestines was examined by ELISA. Significant differences are indicated: * P < 0.05 by Student's *t*-test between each two cohort, n=18 per group. F) The expression level of *Pgk1* was examined in small intestine tissues by quantitative PCR. Significant differences are indicated by Student's *t*-test between each two cohort, n=18 per group.

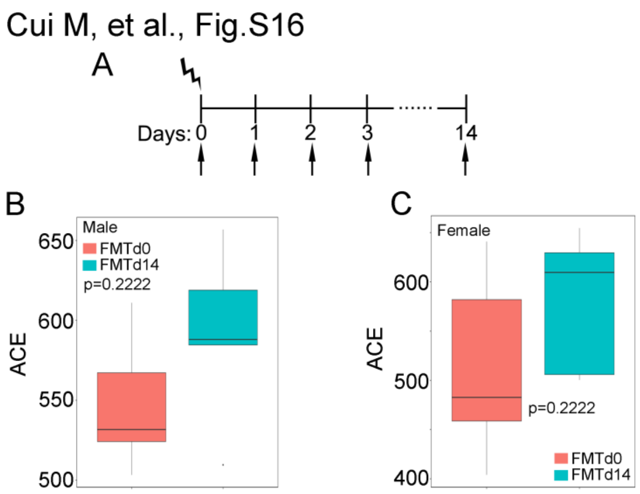

Figure S16. Faecal microbiota transplantation shapes the gut bacterial composition structure. A) Scheme for faecal microbiota transplantation. B, C) The ACE diversity index of intestinal bacteria from male (B) and female (C) was assessed by 16S rRNA high-throughput sequencing after 14 days FMT.

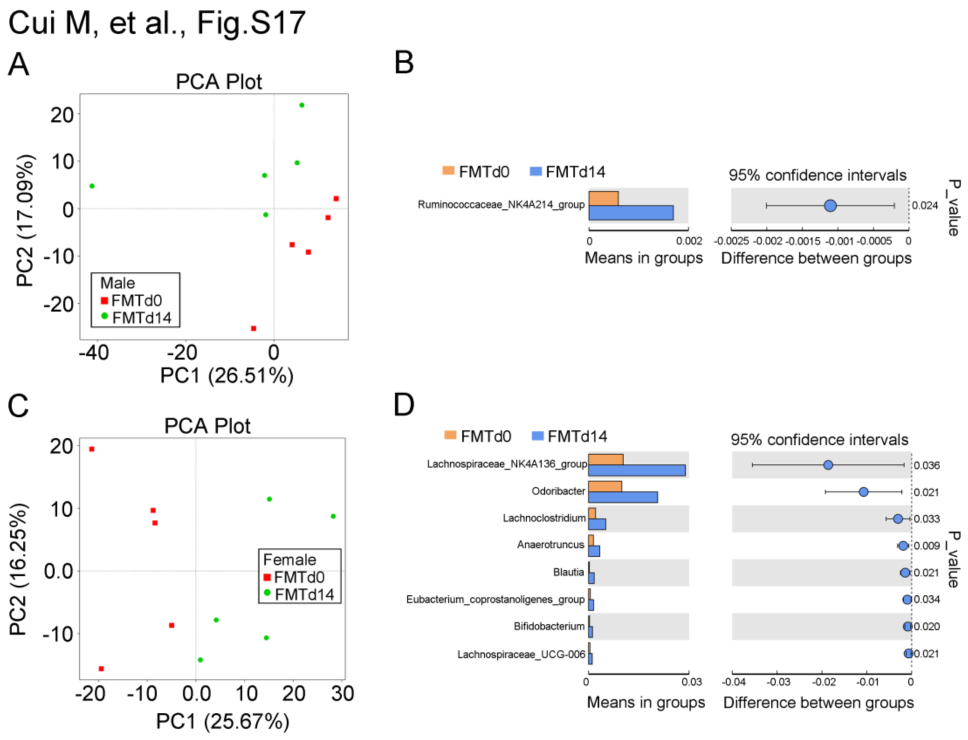

Figure S17. Faecal microbiota transplantation educates the gut bacterial composition pattern. A) PCA was used to measure the shifts in intestinal bacterial composition profile of male recipients after FMT. B) The dominant bacteria at the genus level were showed. Significant differences are indicated: Student’s *t*-test, n = 5 per group. C) PCA was used to measure the

shifts in intestinal bacterial composition profile of female recipients after FMT. D) The dominant bacteria at the genus level were showed. Significant differences are indicated: Student's *t*-test, n = 5 per group.

Supplementary table 1

List of primers used in this paper.

Gene	Primer	Sequence (5′-3′)
**Primers for PCR**		
GAPDH	forward	TGTTTCCTCGTCCCGTAGA
	reverse	CAATCTCCACTTTGCCACTG
TNFa	forward	TTCTCATTCCTGCTTGTGGCA
	reverse	ACTTGGTGGTTTGCTACGACG
Glut1	forward	TATCCTGTTGCCCTTCTGC
	reverse	CCGACCCTCTTCTTTCATCTC
Pgk1	forward	GGAAAACCTCCGCTTTCATGTA
	reverse	GCCTTCTGTGGCAGATTCACA
MDR1	forward	TACGCCTACTATTACACCG
	reverse	CATCAAACCAGCCTATCTC
Nrf2	forward	ACAGTGCTCCTATGCGTGAAT
	reverse	AAGCGGCTTGAATGTTTGTC
**Primers for sequencing**		
515F		GTGCCAGCMGCCGCGGTAA
806R		GGACTACHVGGGTWTCTAAT
